# Gastric cancer genomics study using reference human pangenomes

**DOI:** 10.26508/lsa.202402977

**Published:** 2025-01-27

**Authors:** Du Jiao, Xiaorui Dong, Shiyu Fan, Xinyi Liu, Yingyan Yu, Chaochun Wei

**Affiliations:** 1 Department of Bioinformatics and Biostatistics, School of Life Sciences and Biotechnology, Shanghai Jiao Tong University , Shanghai, China; 2 Department of General Surgery of Ruijin Hospital, Shanghai Institute of Digestive Surgery, and Shanghai Key Laboratory for Gastric Neoplasms, Shanghai Jiao Tong University School of Medicine, Shanghai, China

## Abstract

A pangenome is the sum of the genetic information of all individuals in a species or a population. Genomics research has been gradually shifted to a paradigm using a pangenome as the reference. However, in disease genomics study, pangenome-based analysis is still in its infancy. In this study, we introduced a graph-based pangenome GGCPan from 185 patients with gastric cancer. We then systematically compared the cancer genomics study results using GGCPan, a linear pangenome GCPan, and the human reference genome as the reference. For small variant detection and microsatellite instability status identification, there is little difference in using three different genomes. Using GGCPan as the reference had a significant advantage in structural variant identification. A total of 24 candidate gastric cancer driver genes were detected using three different reference genomes, of which eight were common and five were detected only based on pangenomes. Our results showed that disease-specific pangenome as a reference is promising and a whole set of tools are still to be developed or improved for disease genomics study in the pangenome era.

## Abstract (precis)

Using a concept called graph pangenome for many individual genomes as the baseline can improve the identification and characterization of large-sized genomic variations for disease genomics study.

## Introduction

The availability of reference genomes has been the foundation of genomics research for the past decade. However, as reports of the non-reference sequences and genes continue to increase across a wide range of species, it is becoming increasingly clear that a single genome is insufficient to represent the entire landscape of sequence diversity within a species (Tao et al, 2020). The human reference genome, for example, is currently structured as a linear complex of haplotypes from more than 20 individuals, with 70% of the sequences coming from a single individual. Its framework is biased and erroneous and is not representative of global human genome variation (Wang et al, 2022). For example, identification of structural variants (usually variant length above 50 bps) relies on detecting patterns of discordant read pairs or split read alignments, which in turn depends on the accuracy of read mapping. If reads are too short to cover long repetitive regions of the genome, then assembling and detecting these structural variants is difficult. The limitations of short reads and the bias of the reference genome mean that we may be missing more than 70% of the structural variation in traditional whole-genome sequencing studies (Wang et al, 2022). As a result of the shortcomings of traditional genomes, pangenomes were born.

The concept of pangenomes was introduced in 2005 and has been widely used in bacteria, fungi, plants, and animals (Tettelin et al, 2005; Li et al, 2010; Li et al, 2014; Wang et al, 2018; Li et al, 2019; Sherman et al, 2019; Tian et al, 2020). As the cost of sequencing decreases, the human pangenome is also improving and developing. New sequences ranging from 0.3 to 296 Mb in size have been discovered in different populations (Sherman & Salzberg, 2020). The current pangenomes mainly contain two broad categories: linear pangenomes and graph pangenomes. The linear pangenome contains a traditional reference genome and extra non-reference genome sequences. Most linear pangenomes do not offer the location information of the non-reference sequences, which leads to a result that most aligners simply treat the non-reference sequences as additional sequences tacked onto the genome. In addition, non-reference sequences are obtained by selecting representative sequences, which also lose some or even most of the unique information of individuals. Now the graph pangenome is in a form of new sequences embedded in the reference genome, and the different sequences among individual genomes are represented as new nodes, which keep the positional information of new sequences and the information of each individual. A study demonstrated that whereas graph-based mapping yields higher accuracy than linear alignment on reads that contain known variants, linear genome alignment is superior when the reads do not contain variants (Grytten et al, 2020). At present, the graph pangenome has many applications in the field of genomics study of plants and animals, such as humans, cattle, tomatoes, cucumbers (Hadi et al, 2020; Li et al, 2022; Talenti et al, 2022; Zhou et al, 2022; Liao et al, 2023; Shi et al, 2023).

Currently, the study of the human pangenome in the medical field is still in its infancy. One example of the application of the pangenome to the field of oncology is a previously published study on gastric tumors, which constructed a linear gastric cancer–specific pangenome called GCPan using whole-genome sequencing data from 185 Chinese gastric tumor patients (Yu et al, 2022). In this study, we built a graph pangenome construction pipeline, based on which we constructed the Chinese gastric cancer graph–based pangenome called GGCPan. Then, we performed variant detection based on two Chinese gastric pangenomes (GCPan and GGCPan) and the reference genome GRCh38 in 185 gastric cancer patients. We hope to quantify the effect of different genomes in the disease data, what are the similarities and differences of the results compared with the traditional reference genome-based tumor analysis process, and what are the advantages and disadvantages of each, as well as to find some gastric cancer driver genes that exist only in the Chinese pangenome, to improve the gastric tumor diagnosis and treatment, and even to promote the development of precision medicine.

## Results

### Construction of GGCPan

**Figure S1.**
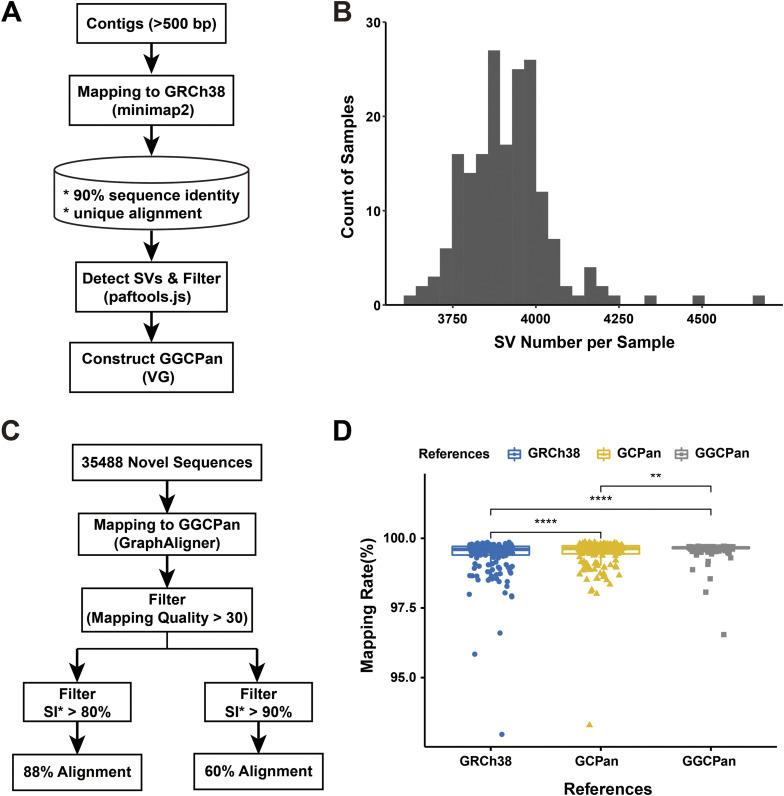
Construction of GGCPan and comparison of mapping rate using three different reference genomes. **(A)** Construction pipeline of gastric cancer graph pangenome GGCPan. **(B)** Histogram of the distribution of the number of SVs detected in the 185 samples. The SVs are detected by paftools.js based on minimap2 alignment results and are applied to construct the GGCPan. **(C)** Pipeline of non-reference sequences of GCPan aligned to GGCPan. **(D)** Read mapping rates of 185 gastric tumor samples on three reference genomes.

We aligned the assembly genome sequences of tumor and normal tissues of 185 patients (contigs of 500 bps or more) to GRCh38, respectively. Then, we extracted the contigs that were aligned to unique positions on the reference genome. Based on the alignment result, we detected 3,632–4,682 structural variants (variant length more than 50 bps) in each sample (Fig S1B). After merging, a total of 39,605 structural variants were detected in the 185 samples. Finally, these variants were embedded into GRCh38 to construct the graph pangenome of gastric cancer samples (see the Materials and Methods section, Fig S1A). We named the gastric cancer graph pangenome as GGCPan.

### Read alignment rate comparison using three reference genomes

We aligned the cancer and paracancer sequencing reads of 185 patients to three reference genomes GRCh38, GCPan, and GGCPan, respectively, and detected SNPs, indels, and SVs based on the alignment results, respectively (see the Materials and Methods section). A downstream comparative analysis was then performed.

We compared the mapping rates of reads aligned to different genomes for 185 patients. We found that using both pangenomes significantly improved the overall mapping rate of reads compared with the results using GRCh38 as the reference (Fig S1D). In particular, the mapping rate of paired-end reads is higher using GGCPan than using GCPan. We believe that it is because we anchor the location of the novel sequence in GGCPan, which avoids the problem of soft cuts and gaps of the reads during the alignment process, and ensure that paired-end reads are aligned at the same position. Although GCPan includes sequences that are not contained in GRCh38, their chromosomal position is unknown. In addition, some non-GRCh38 sequences are highly repetitive. There will be a certain percentage of paired reads aligned to different positions, which will lead to a decrease in mapping quality and affect variant detection.

There are 35,488 non-reference sequences in GCPan. To compare the non-reference sequences in GCPan and GGCPan, we aligned the 35,488 non-reference sequences found by GCPan to GGCPan (Fig S1C). We retained alignment results with a mapping quality greater than 30. Overall, 60% (21,318) of the new sequences could be aligned to GGCPan if we set the sequence identity at 90%, and 88% (31,254) of the new sequences could be aligned to GGCPan if we set the sequence identity at 80%. This suggests that GGCPan contains at least 60% of new sequences detected by GCPan.

### GGCPan has advantages in the detection of structural variants

**Figure S2.**
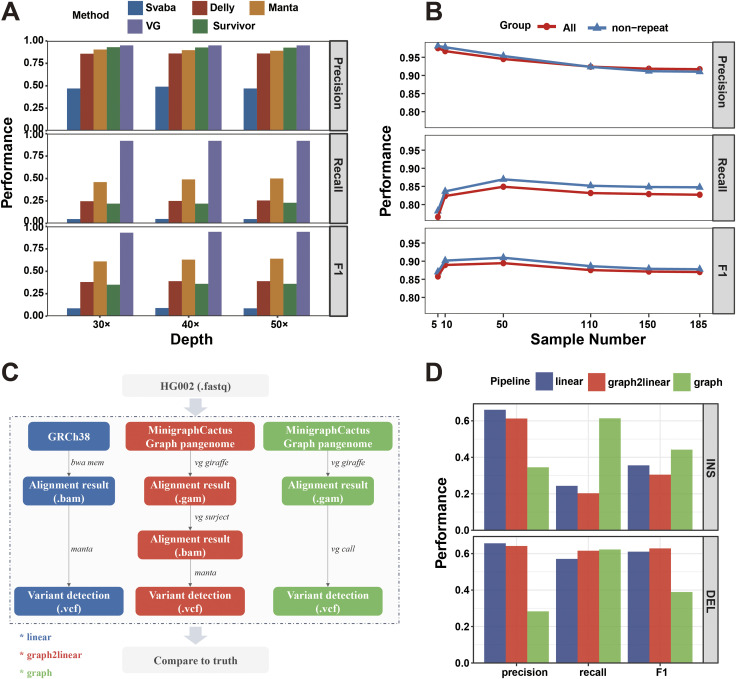
Performance comparison of structural variant detection using three different reference genomes. **(A)** Comparison of the performance of different tools using GRCh38 as the reference for structural variation detection using simulated data with different sequencing depths. **(B)** Effect of the completeness of the graph-modeled pangenome on its performance in detecting structural variants. The x-axis represents the number of samples to construct the graph-modeled pangenome. The five samples used for evaluation were excluded from the samples used to construct the five graph pangenomes. **(C)** Flowchart of the evaluation of different reference genomes and variant identification tools using sequencing data from the GIAB HG002 sample. Different colors represent different identification pipelines. **(D)** Performance evaluation results for variant identification using different reference genomes.

**Figure 1.**
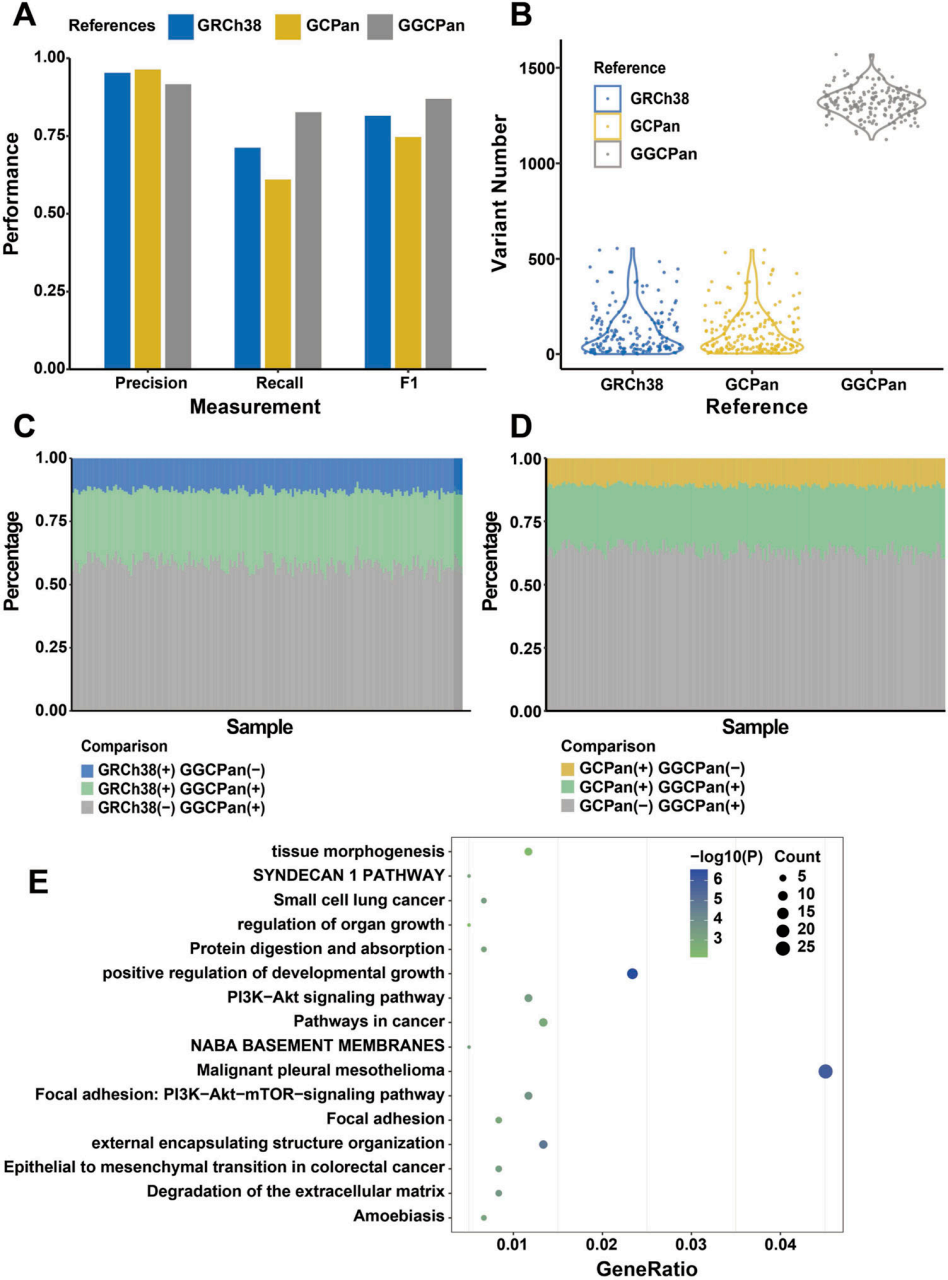
Performance of structural variant detection using three different reference genomes. **(A)** Comparison of the performance of structural variant detection using three different reference genomes in simulated data. **(B)** Number of somatic structural variants detected using three reference genomes in real sequencing data from 185 patients. **(C)** Comparison of SVs detected using GRCh38 and GGCPan in 185 patients. **(D)** Comparison of SVs detected using GCPan and GGCPan in 185 patients. **(C, D)** “+” stands for presence and “−” for absence in (C, D). **(E)** Enriched pathways for SV-related genes. The SVs are detected using GGCPan in 185 samples. The size of the dot represents the number of related genes included in the pathway.

**Figure S3.**
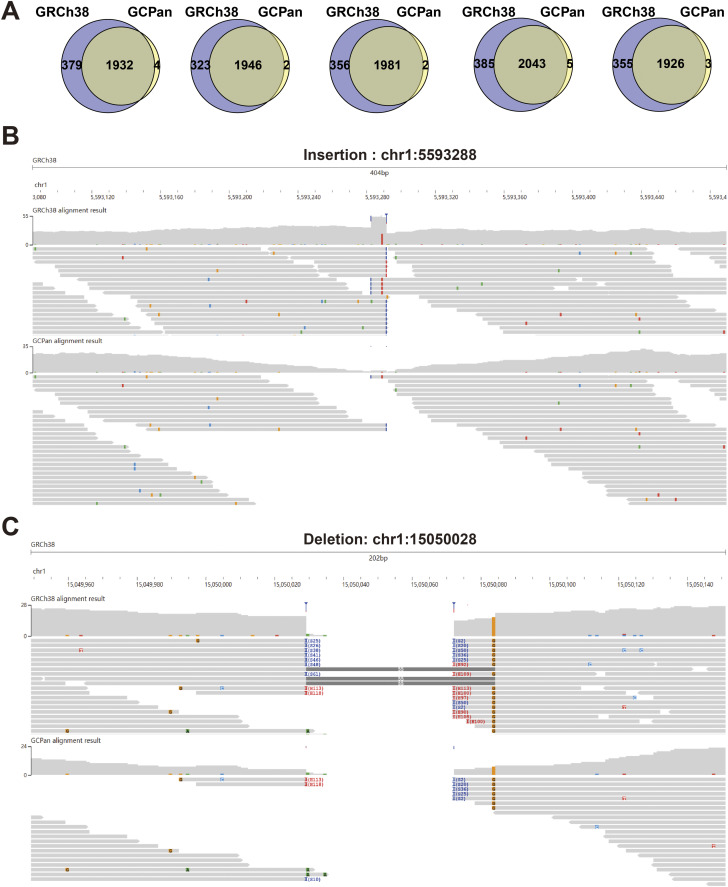
Comparison of structural variants in simulated data (SimuA) using GCPan and GRCh38 as the references. **(A)** Overlap of GRCh38-based SVs and GCPan-based SVs in the five simulated samples (SimuA). **(B)** Example of insertion that was detected in GRCh38 but not in GCPan. **(C)** Example of deletion that was detected using GRCh38 but not using GCPan. **(B, C)** Gray bars in (B, C) represent the alignment of reads at this position when using GRCh38 and GCPan as the reference genomes, respectively.

We want to compare the performance difference in structural variant detection using three different reference genomes. We firstly evaluated the performance of three structural variant detection tools Manta (Chen et al, 2016), Delly (Rausch et al, 2012), and SVaBa (Wala et al, 2018) (Supplemental Data 1, Fig S2A, C, and D, Table S1) (Rausch et al, 2012; Chen et al, 2016; Wala et al, 2018; Kosugi et al, 2019; Hickey et al, 2020, 2024; Zook et al, 2020). The three tools were designed for linear genomes and evaluated well in a previous study (Kosugi et al, 2019). We finally chose one tool that performed best to detect structural variants using linear genomes. We randomly selected five samples from the 185 samples and simulated five whole-genome sequencing samples that contain the structural variants in these five genomes. The reads are paired-end with a sequencing depth of 30× and read length of 150 bps. We named these simulated data as SimuA. We aligned the reads of SimuA to three reference genomes and then detected the structural variants (see the Materials and Methods section). We calculated the mean precision, recall, and f1 values of the five samples (Fig 1A; see the Materials and Methods section). The precisions of GRCh38, GCPan, and GGCPan are 95.30%, 96.34%, and 91.71%, which do not differ too much. GGCPan is slightly lower than the other two linear genomes. The recalls of GRCh38, GCPan, and GGCPan are 71.28%, 61.02%, and 82.70%. The recalls show that GGCPan captures the highest number of true SVs, which is 10–20% more than the other two linear genomes. The recall of GCPan-based SV identification is about 10% lower than that of GRCh38-based. Almost all (99%) of the SVs detected using GCPan are included in SVs detected using GRCh38, and 15% more SVs were detected using GRCh38 as the reference than those based on GCPan (Fig S3A). It might be caused by some reads aligned to the non-reference sequences of GCPan, resulting in a lower number of SVs in the GRCh38 region. Two structural variant examples, an insertion and a deletion, are listed here. For the insertion detected based on GRCh38 rather than on GCPan, more reads were aligned to the location using GRCh38, whereas the same reads were aligned to the non-reference sequences using GCPan as the reference (Fig S3B). The situation of the deletion is similar (Fig S3C). These two examples show that non-reference sequences relative to GRCh38 are insertions, but traditional variant detection tools cannot define non-reference sequences contained in each sample as variants. Based on the evaluation results, we can intuitively see that GGCPan is able to balance accuracy and completeness when detecting structural variants based on short reads, whereas linear genomes miss a lot of true positives, which is also prevalent with other tools (Kosugi et al, 2019). We also performed an evaluation using the GIAB real data and got similar conclusion (Supplemental Data 1) (Hickey et al, 2020, 2024; Zook et al, 2020).

Supplemental Data 1. Evaluation of the impact of different aspects on structural variant detection, including different reference genomes, structural variation identification methods, and the whole-genome sequencing depths.

Table S1. Genotyping evaluation on the Genome in a Bottle dataset from public literature (Hickey et al, 2020).

**Figure S4.**
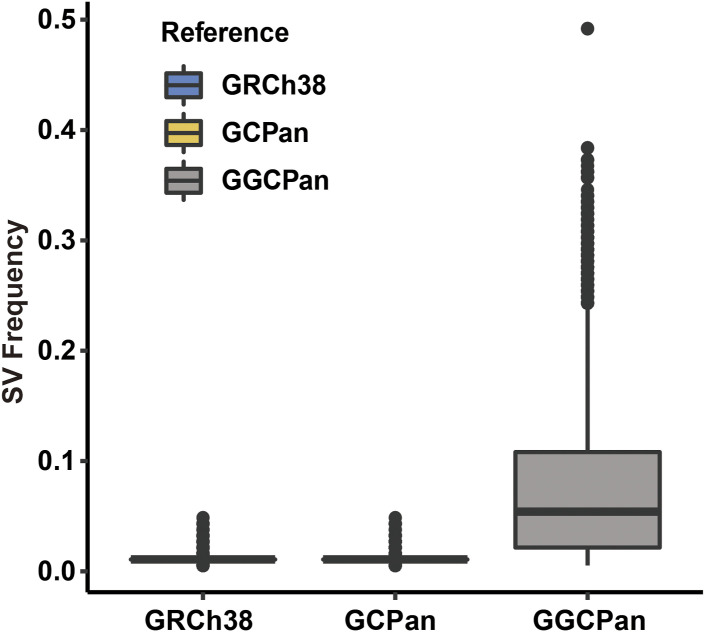
Population frequency of SVs detected in 185 samples using GRCh38, GCPan, and GGCPan.

We detected the structural variants of 185 gastric cancer patients using the three genomes, respectively, and the results were consistent with the simulated data, with 21,415 and 21,367 structural variants detected in the GRCh38- and GCPan-based alignments, respectively, whereas 35,227 structural variants were detected in the GGCPan-based alignment, which was an increase of about 65% (Fig 1B). We compared the overlaps of SVs detected based on GGCPan and the two linear genomes. 26–33% SVs were detected both in GRCh38 and in GGCPan, 9–16% SVs were detected in GRCh38 rather than GGCPan, and 51–65% SVs were detected in GGCPan rather than GRCh38 (Fig 1C). The comparison between GGCPan and GCPan is almost the same (Fig 1D). But less SVs were detected based on GCPan than GRCh38. This suggests that the GGCPan can increase the accuracy and completeness in SV detection compared with GCPan and GRCh38, which is consistent with the findings of the simulation data. Although the linear pangenomes contain non-reference sequences, they are not fit into the traditional variant calling tools. The population frequency of SVs in GRCh38 and GCPan is less than 0.01, which means the SVs are so rare that they just occurred in less than two samples (Fig S4). We further analyzed the correlation of SVs detected based on GGCPan with phenotypes in 185 patients. A total of six phenotypes are listed in the Materials and Methods section. For continuous phenotypic variables, we used the Wilcoxon rank-sum test, and for categorical phenotypes, we used Fisher’s exact test, with the significance thresholds set at *P* < 0.01. There were 1,693 structural variants significantly associated with phenotypes, involving 599 genes. We performed pathway enrichment analysis on these 599 genes and found a series of pathways related to gastric cancer such as “local adhesion,” “protein digestion and uptake,” “pathways in cancer,” “regulation of organ growth,” “tissue morphogenesis” (Fig 1E).

### The completeness of graph pangenome does affect the performance of variant detection

To evaluate the impact of the completeness of graph-modeled pangenome on the detection of structural variants, we randomly selected 5, 10, 50, 110, 150 samples from the 185 gastric cancer samples (excluding the five samples used for the SimuA dataset) and constructed five graph-modeled pangenomes with these samples, respectively (see the Materials and Methods section). We aligned the SimuA samples to the five graph pangenomes and then detected the structural variants. The five samples used to simulate SimuA data were not used to construct the five graph pangenomes. We calculated the mean values of precision, recall, and f1 per graph pangenome. The evaluation is performed using all variants or using only variants within non-repeat regions. The non-repeat regions were constructed by excluding segmental duplications and tandem repeats (using the respective tracks from the UCSC Genome Browser). Sequence alignment was more precise in non-repetitive regions, and therefore, their variant detection is more accurate than in repetitive regions (Fig S2B). However, when the graph pangenome contained more variants, the proportion of repetitive sequences also became larger, and the reads can be aligned to more positions, resulting in greater uncertainty, so the structural variants detected in the non-repetitive regions became fewer instead, and the accuracy also decreased. The precision on all regions decreased from 97.53% to 92.41% when we increased the number of samples for constructing the graph pangenome from 5 to 110. The precision of graph pangenomes constructed using more than 110 samples is almost constant. The tendency of precision shows that using more SVs to construct graph pangenomes does not increase the accuracy of variant detection. By adding in the large numbers of variants, we increase the number of places a read might align and increase the chances that a read might be aligned to an incorrect location. A previous study demonstrated that when 8–12% of known SNPs are included, graph aligners have the fewest number of incorrectly mapped reads (Pritt et al, 2018). However, when the number of variants included is increased beyond that, accuracy declines. The graph pangenome constructed using five samples contained 23.9% of the total number of SVs from the 185 samples. The recall increased from 76.56% to 85.18% when we increased the number of samples for constructing the graph pangenome from 5 to 110. When the sample number to construct graph pangenome is more than 110, the recall line keeps stable. The trend of the recall line suggests that a certain number of known SV catalogs help the NGS data to detect more complete SVs. From the F1 values that balance the precision and recall, using 50 samples to construct the gastric cancer graph pangenome performs best in SV detection.

### There was little difference in the effect of different reference genomes on the detection of small variants, tumor mutation burden (TMB), and microsatellite instability (MSI)

**Figure 2.**
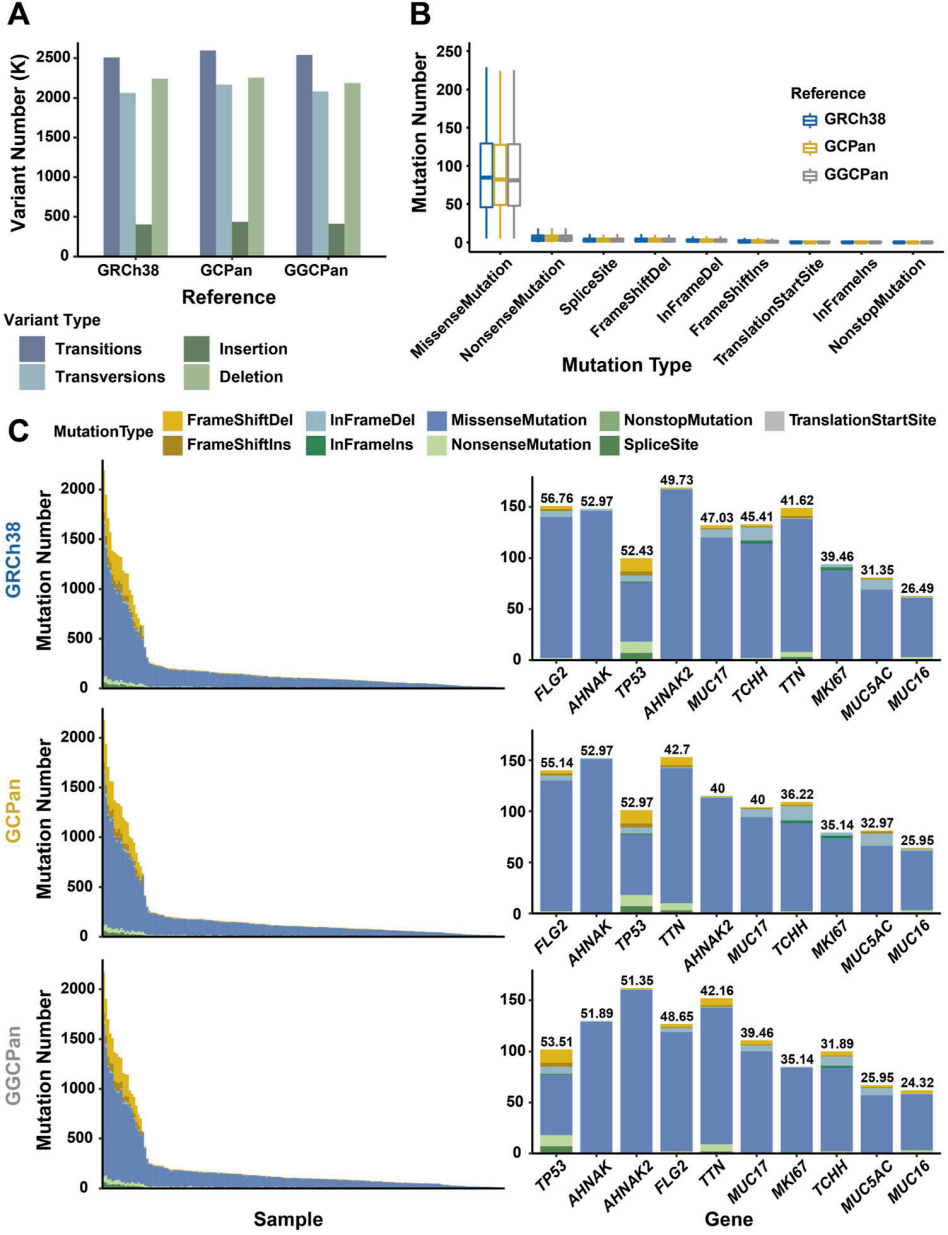
Comparison of numbers and types of small variants detected using three different reference genomes. **(A)** Numbers of SNPs and indels detected in 185 patients with gastric tumors. Transitions and transversions are subtypes of SNPs. Insertion and deletion are subtypes of indels. **(B)** Numbers of different functional types of small variants (SNP, indel) detected based on the three reference genomes. **(C)** Left histograms: numbers and types of small variants (SNP, indel) detected in the three reference genomes in 185 patients; right histogram: types and numbers of variants in genes with mutation rates ranked top 10. The numbers at the top of the histogram represent the mutation rate. Top, middle, and bottom represent results using GRCh38, GCPan, and GGCPan as the reference genomes, respectively.

We detected the SNPs and indels of 185 gastric cancer patients using the GATK (see the Materials and Methods section) and compared the results using three references. We integrated and counted the somatic variants detected on the three genomes for 370 samples from 185 patients and removed redundant variants (Fig 2A). We detected 4,574,851, 4,766,141, and 4,622,619 SNPs and 2,642,387, 2,688,654, and 2,600,457 indels based on GRCh38, GCPan, and GGCPan, respectively. The number of SNPs and indels detected using the three reference genomes did not differ much. Then, we counted the difference in the distribution of the number of different types of mutations using the three reference genomes (Fig 2B). The number of missense mutations is the highest, followed by nonsense mutations, but their numbers using the three reference genomes are almost the same, with no significant difference. We then counted the distribution of the mutation numbers and mutation types in the 185 samples and found that the number of mutations varied greatly among the samples. Some samples contain more than 2,000 mutations, whereas others only contain no more than 10 mutations (Fig 2C). We also counted the number and type of variants in the top 10 genes with the highest mutation rates among the 185 patients (Fig 2C). The top 10 genes were *TP53*, *AHNAK*, *AHNAK2*, *FLG2*, *TTN*, *MUC17*, *MKI67*, *TCHH*, *MUC5AC*, and *MUC16*, which were in slightly different orders using three references. Therefore, in terms of the number of SNPs and indels, mutation types, sample distribution, and gene distribution, the SNPs and indels detected in the three genomes were almost the same.

**Figure S5.**
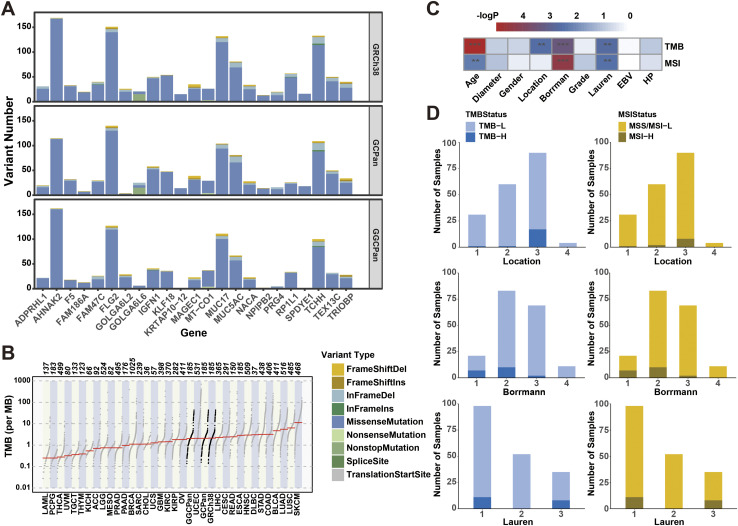
There was little difference among results using different reference genomes on the detection of small variants, tumor mutation burden (TMB), and microsatellite instability (MSI). **(A)** 23 genes with mutation rates differing by more than 5% in 185 samples using the three reference genomes. The y-axis represents the number of mutations per gene in 185 samples. **(B)** TMBs in different cohorts. The three bolded black cohorts are our gastric cancer data using three reference genomes. **(C)** Results of correlation tests between TMB and MSI with each phenotype. Continuous variable phenotypes (e.g., age and tumor diameter) were subjected to Spearman’s correlation test using calculated values of TMB and MSI, and other types of phenotypes were subjected to Fisher’s exact test using state values of TMB and MSI (TMB-H/TMB-L, MSI-H/ MSI-L) for Fisher’s exact test. Each grid color corresponds to the negative logarithmic value of the *P*-value of the correlation test. In the figure, “*” indicates that the *P*-value is between 0.05 and 0.01, “**” indicates that the *P*-value is between 0.01 and 0.001, and “***” indicates that the *P*-value is less than 0.001, and unlabeled positions indicate that the correlation is not significant. **(D)** Sample distribution of TMB-H, TMB-L and MSI-H, MSI-L/MSS in the subtypes of location, Borrmann, and Lauren.

The SNPs and indels detected based on the three genomes were largely consistent. However, when we focus on the mutation rate, mutation site, and number of mutations in a specific gene, the reference genome and pangenome analysis methods may show significant differences. There were 23 genes with mutation rates differing by more than 5% using the three genomes (Fig S5A). Among them, the mutation rates of the genes based on the two pangenomes were generally smaller than those based on GRCh38. It was because some of the reads were aligned to non-reference sequences in the pangenomes, thus resulting in a lower mutation rate. This also reflects the incompleteness of GRCh38; that is, there are many non-reference sequences that contain information, which has been omitted because of the limitation of the reference genome. Four of these 23 genes, *ADPRHL1*, *F5*, *SPDYE1* and *MUC17*, were subsequently detected as candidate driver genes (Figs S5A and 3A).

**Figure 3.**
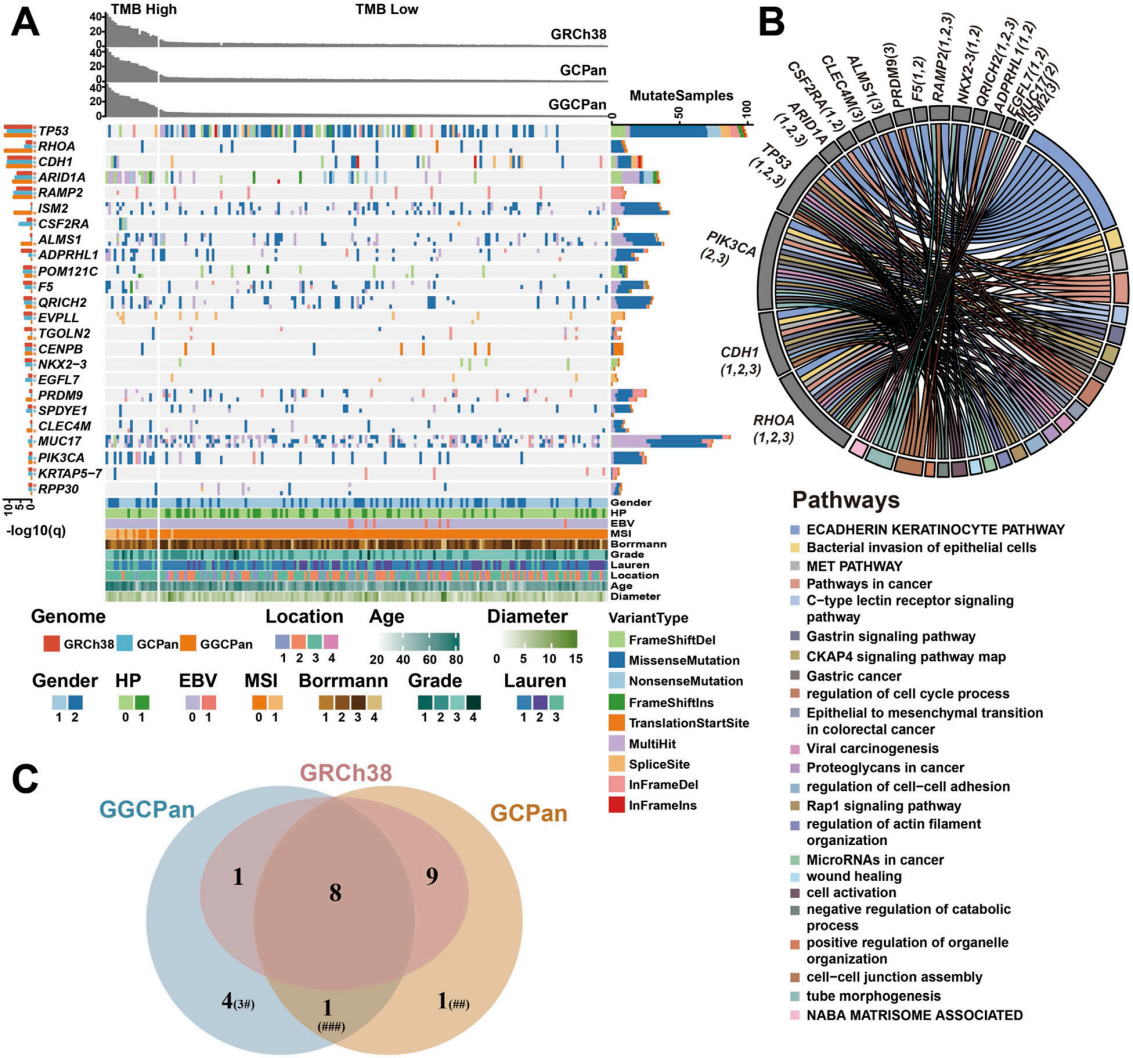
Comparison of candidate driver genes detected using different reference genomes. **(A)** Candidate driver genes of gastric cancer detected using the three reference genomes. The left bar graph shows the −log_10_(q) value of each gene, and the “*” next to a gene name indicates that the gene was determined as a driver gene using this reference genome. The q-value here stands for the significance of the gene being identified as a driver gene. The right bar graph represents the number of mutations and mutation types for each gene. The upper bar graph represents the TMB values of each sample using the three different reference genomes. **(B)** Enriched pathways related to the candidate driver genes. The significance threshold for enrichment analysis was *P* < 0.05. Numbers in parentheses represent that the gene was identified as a driver gene using the corresponding reference genome. “1” represents GRCh38, “2” represents GCPan, and “3” represents GGCPan. **(C)** Overlap of the candidate driver genes detected using the three reference genomes. “#” indicates that three of the four genes are related to cancers in previous studies. “##” indicates that this gene is related to cancers in previous studies. “###” indicates that this gene is related to cancers in previous studies.

TMB is the number of somatic non-synonymous mutations in a given genomic region, usually expressed as the number of mutations per mega-base (mut/Mb). TMB can indirectly reflect the ability and degree of neoantigen production by tumors and predicts the efficacy of immunotherapy for a variety of tumors. We compared the differences in TMB when using three different reference genomes (see the Materials and Methods section, Table S2). There was no significant difference between the TMBs using the three reference genomes (Wilcoxon’s test, *P* > 0.1). Comparing the TMB values using the three genomes with TCGA results (Fig S5B), there is no significant difference between our results and TCGA gastric cancer cohort (STAD), which verifies the accuracy of the results of our sequencing, sequence alignment, and variant detection. In TCGA analysis on gastric tumors (Cancer Genome Atlas Research Network, 2014), researchers used TMB = 11.4 as the threshold to distinguish hypermutated samples from conventional mutated samples, so we labeled 166 samples of gastric tumor samples with TMB values less than 11.4 as TMB-L, and 19 samples with TMB values greater than or equal to 11.4 as TMB-H. The TMB status classification of 185 gastric tumor samples was completely consistent using the three reference genomes. The proportion of hypermutated samples to the total number of samples was 10.27%.

Table S2. Tumor mutation burdens of 185 samples using three genomes.

MSI is the insertion or loss of base pairs in microsatellite regions because of replication errors. MSI was firstly identified in colorectal cancer and is thought to be a feature of hereditary nonpolyposis colorectal cancers, and since then, it has been found in a variety of sporadic tumors (e.g., gastric, lung, endometrial). We calculated and validated MSI fragments for 185 patients using each of the three genomic contexts (see the Materials and Methods section) and labeled 11 of the 185 samples as MSI-H and the remaining 174 samples as MSI-L or MSS. The MSI status classification of 185 samples was completely consistent using the three reference genomes. We validated the MSI loci in 185 samples with five biomarkers in the Bethesda panel (Boland et al, 1998), which is commonly used for MSI identification (see the Materials and Methods section). Next, we compared the consistency of MSI status determination based on NGS alignment results and the Bethesda panel (see the Materials and Methods section). The consistency of three references and the Bethesda panel is the same (0.95) (Table S3). This indicates that the NGS results aligned to pangenomes are in the same effect with GRCh38 in determining the MSI status.

Table S3. Calculation of kappa values using different references.

Because the Bethesda panel was originally designed for colorectal cancer patients, it may not be fully applicable to gastric tumor patients. Therefore, we wanted to find MSI-H biomarkers for gastric tumors based on the alignment results of the 185 patients. Because the results of three genomes were identical in determining the MSI status of the 185 samples, we chose the results of only one of them to explore the new biomarker; here, we chose the GCPan. We found six shared MSI loci in the MSI prediction results of 11 MSI-H patients, which appeared in no more than one of the 174 MSI-L patients. We designed PCR primers for the two shared MSI loci, and the other four loci were too highly repeated to design primers (Table S4). We inferred the 2 MSI loci as potential MSI-H markers, which are single-base repeats on chromosomes 2 and 8, respectively. We jointly analyzed these two potential markers and the five biomarkers in the Bethesda panel and found that the results obtained from the combination of our two potential markers and the BAT-25 and BAT-26 loci in the Bethesda panel in determining the MSI status were in agreement with the NGS results up to 1 (Table S3). Therefore, we believe that the combination of our two MSI loci plus two loci, BAT-25 and BAT-26, to determine the MSI status of patients with gastric tumors is more effective than the traditional Bethesda panel.

Table S4. Primers of the candidate MSI-H markers.

Because there was little difference between MSI and TMB detected using the three reference genomes, we performed correlation analyses with phenotypes using GRCh38-based results. The test showed a moderate correlation between the actual values of MSI and TMB (Spearman’s rank correlation coefficient, r = 0.59) and a strong association between MSI status classification and TMB status classification (Fisher’s exact test, *P* = 9.59 × 10^−11^). In addition, TMB and MSI were significantly correlated with age, Borrmann typing, and Lauren typing (Fig S5C and D). Patients with TMB-H and MSI-H were more likely to develop cancer in the gastric antrum (sinus), where TMB correlated significantly with the location of cancer development (*P* = 0.0026); TMB-H (7/14) and MSI-H (6/15) were more likely to be found in type I tumors with Borrmann staging and showed significant association with TMB (*P* = 0.0005) and MSI (*P* = 5.17 × 10^−5^); according to Lauren typing, there were no samples with TMB-H or MSI-H in diffuse tumors and Lauren typing showed statistically significant association with both TMB (*P* = 0.0024) and MSI (*P* = 0.0036).

### A more comprehensive set of candidate driver genes was detected using the two pangenomes compared with GRCh38

We used the 166 TMB-L samples for driver gene prediction (see the Materials and Methods section), because somatic hypermutations are induced by different mechanisms than conventional mutations, and past studies have shown that extremely high mutation rates in hypermutated samples can severely affect the analysis results (Cancer Genome Atlas Research Network, 2014). A total of 24 candidate cancer driver genes were detected using the three reference genomes, of which 18 were detected using GRCh38, 19 using GCPan, and 14 using GGCPan. There were eight genes that were determined to be candidate driver genes using all three reference genomes, which were *TP53*, *CDH1*, *RAMP2*, *ARID1A*, *POM121C*, *RHOA*, *QRICH2*, and *CENPB* (Fig 3A and B). The mutation types, the number of mutations, and the population mutation rates of the eight genes using the three reference genomes were essentially the same. Four genes were identified as driver genes only by GGCPan as the reference (Fig 3C). These four genes were double-checked (Supplemental Data 2, Table S5).

Supplemental Data 2. Manual check of the four driver genes detected only by GGCPan.

Table S5. MutSigCV output reports of the four driver genes.

Of the 24 candidate driver genes, 16 genes are recorded in the Network of Cancer Genes (NCG) (Repana et al, 2019) or reported in cancer-related studies (Table S6) (Liang et al, 2004; Yue et al, 2007; Cancer Genome Atlas Research Network, 2014; Houle et al, 2018; Vojkovics et al, 2018; Repana et al, 2019; Yang et al, 2019; Aaltonen et al, 2020; Li et al, 2020; Liu et al, 2020; Tinholt et al, 2020; Yu & Gao, 2020; Weng et al, 2021; Choi et al, 2023). There are nine candidate driver genes that are detected based on GRCh38 and GCPan but not on GGCPan. And six of the nine genes were related to cancers (Table S6). Gene *PIK3CA* was detected based on GCPan and GGCPan but not on GRCh38, and it was reported as a gastric cancer driver gene in TCGA-STAD (Cancer Genome Atlas Research Network, 2014) and Stomach-AdenoCA (Aaltonen et al, 2020) cohorts. *ISM2*, *ALMS1*, *PRDM9*, and *CLEC4M* were detected as candidate driver genes only based on GGCPan. And three of these four genes were related to cancers (Table S6). *MUC17* was detected only based on GCPan and was related to gastric cancers (Table S4). We performed pathway enrichment analysis of these 24 candidate driver genes, 17 of them were enriched in gastric cancer–related pathways such as “ECADHERIN KERATINOCYTE PATHWAY,” “Bacterial invasion of epithelial cells,” “Gastric cancer,” “Pathways in cancer” (Fig 3B). Seven genes (*POM121C*, *EVPLL*, *TGOLN2*, *CENPB*, *SPDYE1*, *KRTAP5-7*, and *RPP30*) were not enriched in functional pathways.

Table S6. Information of the 24 candidate driver genes.

Among these 24 genes, 11 genes had mutation rates greater than 5% using all three reference genomes (the number of patients with mutations in this gene was greater than 9, Table S7), and we considered these genes most likely to be tumor driver genes. The mutation rates of *TP53* (52.43%, 52.97%, and 53.51% in the three genomes, respectively) and *MUC17* (47.03%, 40%, and 39.46% in the three genomes, respectively) were the highest and much higher than those of the other genes. *ADPRHL1*, *POM121C*, *QRICH2*, *SPDYE1*, and *ISM2* are genes that have high mutation rates in our cohort, but no association with cancer has been reported yet.

Table S7. Mutation rate of significant mutated genes.

GCPan and GGCPan have covered all the 24 candidate driver genes. Furthermore, four candidate driver genes were detected based on GGCPan, three of which have been documented in the literature or databases as being associated with cancer or enriched in gastric cancer–related pathways. One gene (*MUC17*) that is only detected based on GCPan was also related to gastric cancer. This demonstrates the completeness of the pangenomes in detecting driver genes and its substitutability for GRCh38.

**Figure S6.**
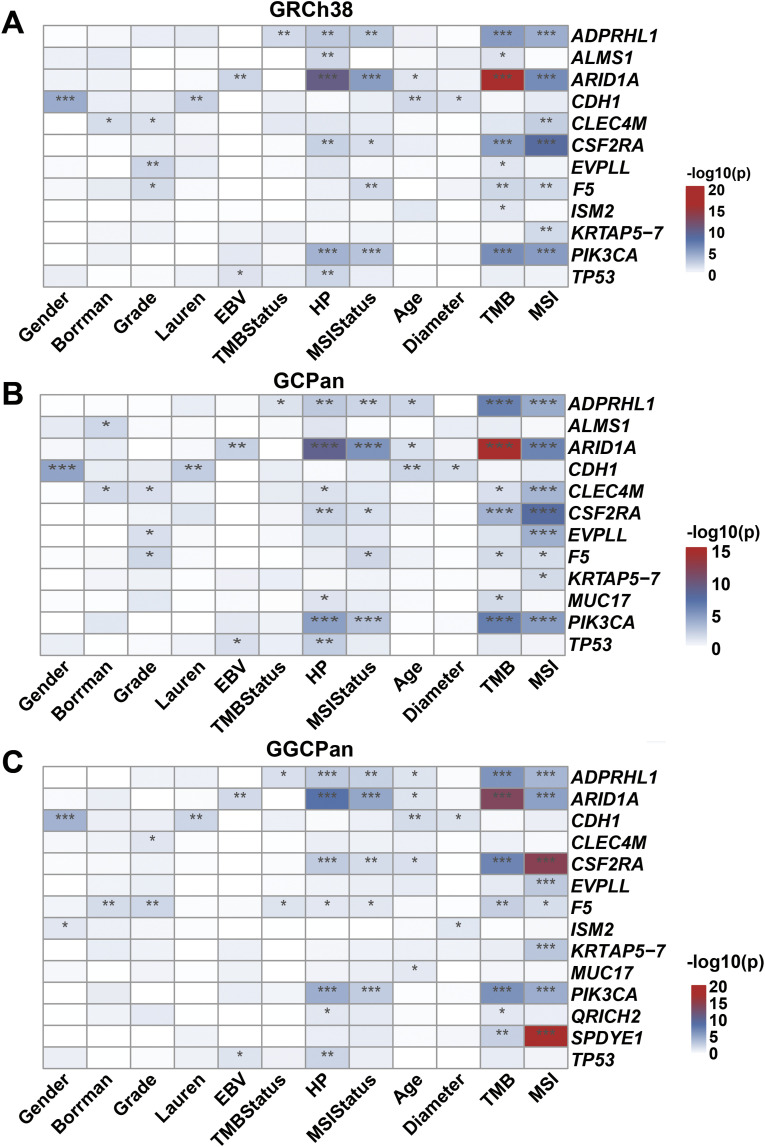
Correlation between the mutation status of these 24 significantly mutated genes in the cohort (yes/no mutation) and the clinical phenotype of the 185 patients. **(A)** Genes significantly related to phenotypes using GRCh38. **(B)** Genes significantly related to phenotypes using GCPan. **(C)** Genes significantly related to phenotypes using GGCPan. In the figure, “*” indicates that the *P*-value is between 0.05 and 0.01, “**” indicates that the *P*-value is between 0.01 and 0.001, and “***” indicates that the *P*-value is less than 0.001, and unlabeled positions indicate that the correlation is not significant.

We analyzed the correlation between the mutation status of these 24 genes in the cohort (yes/no mutation) and the clinical phenotype of the patients, where for binary variable phenotypes, we used Fisher’s exact test, and for continuous variable phenotypes, we used a point-biserial correlation test (Fig S6A–C). A total of 17 genes were significantly associated with the phenotype. The genes significantly associated with gender were *CDH1* and *ISM2*, with female patients more likely to have *CDH1* mutations. Genes significantly associated with age were *ADPRHL1*, *ARID1A*, *CDH1*, *CSF2RA*, and *MUC17*, with older patients more likely to have mutations in *ARID1A*, *ADPRHL1*, and *MUC17*. *TP53* mutations were significantly associated with EBV-negative patients. *ADPRHL1* mutations were significantly associated with HP-negative patients. *CDH1* was significantly associated with tumor diameter, and patients with *CDH1* mutations had larger tumor diameters. *CLEC4M* and *F5* were significantly associated with the gastric cancer grade, and mutations in *ISM2* and *F5* were more likely to be found in well-differentiated tumors. *CDH1* was significantly associated with Lauren staging, and gastric tumors with *CDH1* mutations were more likely to be identified as diffuse tumors. In addition, the genes *ADPRHL1*, *ARID1A*, *CSF2RA*, and *PIK3CA* were significantly correlated with all or part of MSI and TMB, and mutations in these genes were more likely to be found in patients with high levels of TMB and MSI.

### Comparison of candidate driver genes with gene PAV analysis results

**Figure 4.**
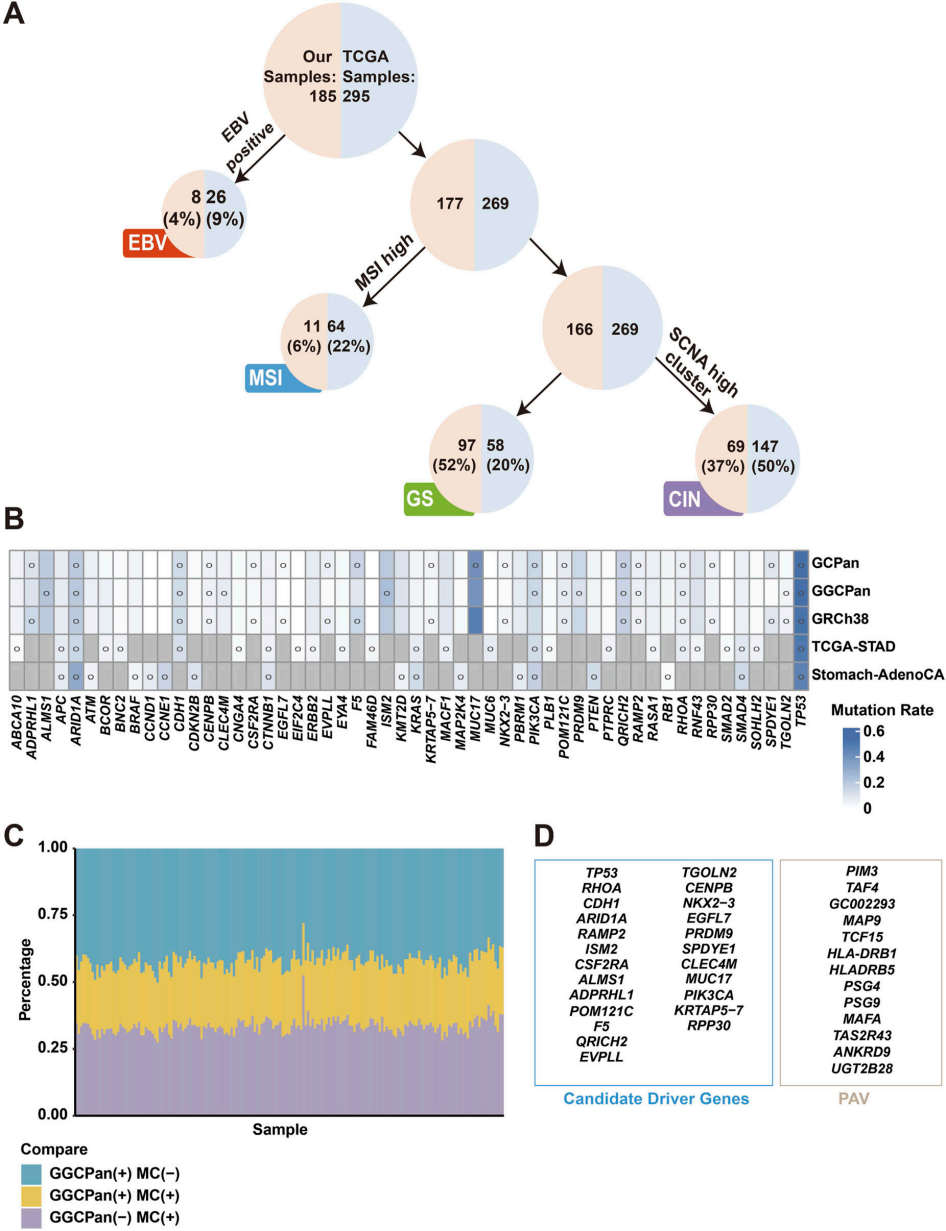
Comparison of molecular subtypes, candidate driver genes, and structural variations with previous studies. **(A)** Decision trees of molecular subtypes of the 185 gastric cancer patients and TCGA-STAD samples. **(B)** Comparison of candidate driver genes detected using three reference genomes in the 185 samples and those from two different gastric cancer cohorts (TCGA-STAD and Stomach-AdenoCA). The blue color represents the mutation rates of genes in each cohort. The gray color represents unknown mutation rate information for the gene. A circle indicates that the gene was determined to be a driver gene in this cohort. **(C)** Comparison of structural variants detected using GGCPan and MC, a graph pangenome constructed with healthy samples. “+” stands for presence and “−” for absence. **(D)** There is no overlap between the 24 candidate driver genes and the genes found to be significantly associated with the phenotype by GCPan PAV analysis.

We also compared the 24 candidate driver genes with the genes significantly associated with phenotypes by GCPan PAV analysis in a previous study and found no intersection (Fig 4D). This suggests that the traditional driver gene identification method and the pangenome-based association analysis of gene PAV and phenotype are more complementary. In terms of methodological comparison, the driver gene prediction process described above focuses on mutations within genes, especially in exon regions, mainly including single-nucleotide mutations and insertion and deletion mutations in small segments, in addition to the mutation rate of genes in the population. The pangenomic PAV analysis, on the other hand, focuses on the presence and absence of genes and large nucleotide sequences, as well as the association between the presence and absence of these genes and the clinical phenotype of the patient. The two methods have different focuses and different data, and naturally predict very different genes. We note that among the 24 candidate driver genes, only three genes were associated with Borrmann typing and no genes were associated with tumor location. However, of the 13 phenotypically associated genes obtained by PAV analysis, six genes were significantly associated with Borrmann typing, two genes were significantly associated with tumor location, and no genes were associated with sex, age, or *H. pylori* infection. These two analyses showed some complementarity in phenotypic associations, but whether there is an intrinsic association needs to be revealed by further studies.

### Comparison with two gastric cancer cohorts

**Figure S7.**
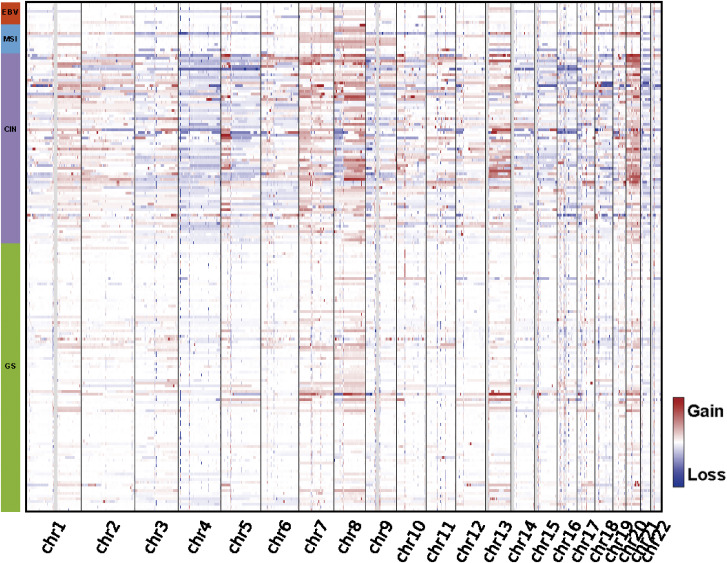
Differences in somatic copy-number variants of the four subtypes, with red color representing copy-number amplification and blue color representing copy-number deletion. The color bar on the left represents the division of the 185 samples into four subtypes. The heatmap represents the copy-number variation for each sample. The red color represents copy-number amplification, and the blue color represents copy-number deletion.

According to the criteria of molecular subtypes of gastric cancer delineated by TCGA (Cancer Genome Atlas Research Network, 2014), we classified our 185 samples into four subtypes, EBV (4%), MSI (6%), GS (52%), and CIN (37%), according to the decision tree method (Fig 4A; see the Materials and Methods section). It can be found that the proportion of EBV-positive patients and MSI-H patients are both lower than that of TCGA sample, whereas the proportion of GS subtypes is more than twice that of TCGA sample, which indicates the difference between the composition of our sample and that of TCGA sample. This finding suggests that our cohort may have found a lot of new information that was not found in either of the two existing cohorts. After categorizing the 185 samples into four subtypes, we compared the somatic copy-number variation on chromosomes 1–22 for each sample (Fig S7). The copy-number amplifications and deletions are more pronounced in the CIN subtype than in the other three subtypes, which is consistent with TCGA results.

The candidate driver genes predicted in this study were analyzed in a comprehensive comparison with the driver genes reported in TCGA Stomach Cancer Cohort (TCGA-STAD) (Cancer Genome Atlas Research Network, 2014) and the driver genes reported in the ICGC/TCGA pancancer analysis for mutations in the stomach cancer cohort (Stomach-AdenoCA) (Aaltonen et al, 2020) (Fig 4B). *TP53* showed a high mutation rate in all cohorts and was predicted as a significant driver gene; the other gene predicted as a driver gene by all cohorts was *ARID1A*. *PIK3CA* was reported to be a significant driver gene in both pangenomic cohorts, TCGA-STAD and Stomach-AdenoCA, but the result on the GRCh38 cohort in this experiment did not show significance because of the threshold setting (q = 0.133). *KRAS*, *APC*, and *SMAD4* were reported as driver genes in the two published cohorts but no significant results in our two cohorts, where the q-value of *SMAD4* was less than 0.3 in all three of our cohorts (Table S6), but its *P*-value was less than 0.001, which was not significant because of the threshold setting. *KRAS* and *APC* had low mutation rates in the samples of our experiment. There were also several genes including *CCNE1*, *FAM46D*, and *SMAD2* that were not determined as driver genes because of low mutation rates in the samples of this experiment. Based on the results of this study, 19 candidate driver genes that were not reported in previous studies were detected. In addition, there were several zinc finger protein–related genes reported as driver genes in the GRCh38 cohort and the GCPan cohort, but because of the excessive number of repetitive sequences within their genes, we considered the detection of such genes based on the short-read-length sequencing data to be unreliable, and therefore, we filtered out these genes.

### Comparison of structural variant identification results using GGCPan and Minigraph-Cactus pangenome suggests the huge impact of reference graph pangenome

In 2023, the Human Pangenome Reference Consortium (HPRC) released three graph pangenomes that are constructed based on 47 healthy human samples. Two of them contain both small variants (SNPs/indels) and structural variants. The remaining one pangenome called the Minigraph-Cactus graph pangenome (MC) (Liao et al, 2023) contains only structural variants. We aligned the sequencing data from 185 samples to this healthy human graph pangenome and detected structural variants in each sample. We compared SVs detected based on this MC graph pangenome and our gastric tumor graph pangenome GGCPan (Fig 4C) and found that the percentage of overlapping SVs found using two pangenomes was low and each of the two graph pangenomes detected 30–50% private SVs. The numbers of SVs detected based on MC in the 185 samples were 11,326–23,701, whereas the numbers were 10,696–16,923 if GGCPan was the reference, and the numbers of common SVs identified using the two reference genomes were 4,638–6,413. A greater percentage of private SVs were detected in GGCPan, and these SVs were specific to patients with gastric tumors. In contrast, the SVs identified in the MC graph pangenome occurred mostly in healthy individuals and were not associated with gastric cancer. These results indicated the necessity of constructing disease-specific pangenomes.

### Time and memory used with three reference genomes

We compared the time and memory used when we did the analysis pipeline using the three reference genomes (Tables 1 and 2). GRCh38 and GCPan are both linear genomes, and the analysis tools are the same. The non-reference sequences in GCPan make it a little longer in alignment, preprocessing, and variant calling. The time used with GGCPan is much less compared with the two linear genomes. On the contrary, the memory used with GGCPan is much bigger than GCPan and GRCh38. This is a common phenomenon in the field of graph-based pangenomes.

**Table 1.** Time required to analyze each sample using three different reference genomes (clock hours).

Reference	GRCh38	GCPan	GGCPan
Construct	—	<5	3
Alignment	7	7.5	2
GATK preprocess	12	13	12
SNP and indel detect	6	7	6
Structural variant detect	2	2.5	3

“—”: not needed. Only the time and memory requirement after genome assembly was constructed.

**Table 2.** Memory for each sample using three reference genomes (GB).

Reference	GRCh38	GCPan	GGCPan
Construct	—	<200	400
Alignment	40	40	60
GATK preprocess	40	40	12
SNP and indel detect	4	4	4
Structural variant detect	28	28	320

“—”: not needed. Only the time and memory requirement after genome assembly was constructed.

## Discussion

Graph pangenome has a great range of application fields including animal and plant genomic study. However, in the disease genomics area, to the best of the authors’ knowledge, this is the first study using graph-based pangenome. We systematically compare the effectiveness of reference genomes including GRCh38 and different forms of pangenomes in disease genomics study. In terms of read mapping rate, both pangenomes significantly improved the read mapping rates. Among them, the GGCPan (graph pangenome) has a slightly higher mapping rate than the GCPan (linear pangenome), which indicates the shift of the pangenome from a linear to a graphical model improves the capability to represent the genetic diversities in a population. The analysis in this study illustrated that the three reference genomes did not differ much in detecting small variants. The main difference is reflected in the detection of SVs using NGS data. GGCPan detected more true SVs compared with the linear genome, which was due to the sequence integrity and positional integrity of GGCPan. However, adding too many variants not only increases the number of positions to align, but also increases the chance that a read may be aligned to a wrong position. Therefore, the preprocessing of variants is important. Currently, there are some drawbacks to construct graph pangenomes by embedding variants into the reference genome. Taking structural variants as an example, it is difficult to balance the total number of variants and the degree of duplications when merging and removing redundancy of structural variants from different samples. Another issue is the file format for variants. The common file format for variants is the vcf format, which has limited expressive power and is particularly difficult to deal with different variants at the same position in different samples, which often differ by only a few bases in position but represent insertions or deletions of large segments. There, variations cannot be simplified to SNPs or indels. In addition, in this study, we only used a method to detect known variants included in the graph pangenome. If we apply this pangenome to a new dataset, novel variants not included in this graph pangenome are likely to be missed or incorrectly detected by the graph pangenome. This is a significant limitation of the current graph pangenome–based analysis. However, both graph pangenomes and linear pangenomes are superior to or equal to GRCh38 in terms of read mapping rate and small variant detection.

Using a linear pangenome is similar to using the traditional human reference genome, which means a wide range of tools are available. However, the tools using a graph pangenome as the reference are still under development and are incompatible with many tools using linear genomes as the reference. In this study, during the evaluation we had to convert graph pangenomes to a file format compatible with linear genomes, in which some genomic diversity information was lost. In addition, the memory required in construction and variant detection using graph pangenome was much higher compared with linear genomes although the time requirement was reduced.

In terms of driver genes, we detected a total of 24 driver genes using three different references, and no gene was detected only using the GRCh38 as the reference, which indicates that using the two pangenomes can cover the results of using the human reference genome GRCh38. Also, because of the current variant annotation databases, our comparisons were all limited to genomic regions common to both pangenomes and GRCh38. Novel sequences in the pangenomes, on which novel genes were also predicted, were not compared in this study. Though, it is possible that driver mutations occur in these regions.

In conclusion, there is little difference in using three different genomes as the reference for the detection of small variants and MSI status determination. Using pangenomes as the reference genome performs better than using the human reference genome GRCh38 for SV detection and driver gene detection. Pangenomes also improve the short-read mapping rate. Using graph pangenome as the reference genome might become the trend of disease genomics study. However, a whole set of new tools are still to be developed or improved.

## Materials and Methods

The experimental material information involved in this project is mainly cancer patient samples and sequencing data–related information. The analysis methods include a variant detection process based on pangenomes and the human reference genome, and a workflow for gastric tumor analysis using the results of variant detection designed with reference to TCGA’s method.

### Sample information

All samples were diagnosed with gastric cancer and underwent gastrectomy at Ruijin Hospital of Shanghai Jiaotong University School of Medicine (n = 140) and Shanghai Cancer Center of Shanghai Medical College of Fudan University (n = 50). All patients did not undergo any neoadjuvant or adjuvant chemotherapy and radiotherapy before surgery. Informed consent was obtained from all participating patients. Cancerous tissues and non-cancerous mucosa more than 5 cm from the main tumor were collected within 30 min after surgery, immediately frozen in liquid nitrogen, and stored at −80°C until DNA and RNA were extracted. All enrolled cancerous tissues showed a purity of 70% of tumor cells. Each sample contained six phenotypes, which were age, gender, Borrmann, Lauren, grade, and location.

### Whole-genome sequencing

Genomic DNA was extracted from patient tissues using the QIAamp DNA kit (QIAGEN), and sequencing libraries were constructed using TruSeq DNA LT Sample Preparation Kit V2 (Illumina) according to the protocol provided by the manufacturer. After purification, quantification, and validation of the DNA libraries, they were sequenced on an Illumina sequencing system (HiSeq X10) according to the manufacturer’s double-ended (2 × 150 bps) protocol. Because of the genotypic mismatch between the primary tumor tissue and the corresponding non-cancerous gastric mucosa, five pairs of samples were removed, yielding 185 pairs of samples for further analysis. Raw Illumina reads were processed for quality control using FastQC (http://www.bioinformatics.babraham.ac.uk/projects/fastqc/).

### Construction of GGCPan

The assembled cancer and normal contigs (500 bps or more) from 185 gastric tumor patients were aligned to GRCh38, respectively, using minimap2 (2.23-r1111) (Li & Birol, 2018) with the parameter “*-a -x asm10*.” Based on the alignment results, the contigs that were not aligned to the unique position were filtered out, and then, structural variants were detected using paftools.js, a built-in module of minimap2. A total of 39,605 SVs were detected in the 185 patients. These SVs were then embedded into GRCh38 using the autoindex module of VG (v1.43.0) (Sirén et al, 2020) to construct the graph-modeled pangenome, called GGCPan, with the parameters “*vg autoindex -workflow giraffe -r -v -t -p*.”

### Sequencing read alignment

Whole-genome sequencing reads of 185 patients were aligned to GRCh38 and GCPan using BWA-MEM (0.7.17) (Li, 2013 *Preprint*) with default parameters, and the alignment results were recorded in bam format. Whole-genome sequencing reads of 185 patients were aligned to GGCPan using the giraffe module (Sirén et al, 2021) of VG with the parameter “*vg giraffe -t -Z -m -d -x -f -N*,” and we got the graph-based alignment result (.gam). Then, the graph-based alignment result was remapped to GRCh38 using the surject module of VG with the parameter “*vg surject -x -b -P -i*” to transfer the alignment result to bam format that is compatible with tools that are commonly used to analyze linear genomes. PCR repeats were labeled using the MarkDuplicates module of GATK. Base quality recalibration based on gold-standard datasets of single-nucleotide polymorphism (SNP) and insertion and deletion (indel) mutations was conducted using the BQSR module of GATK.

### Variant detection

Somatic SNP and indel mutations were detected with the MuTect2 module of GATK (default parameter) using GRCh38, GCPan, and GGCPan, respectively. Structural variants based on GRCh38 and GCPan were detected using Manta (v1.6.0) (Chen et al, 2016). Structural variants based on GGCPan were detected using vg call with parameters “*vg pack -x -g -Q 5 -s 5*” and “*vg call -k -a*.” SNPs and indels were annotated using vcf2maf (v1.6.19) (https://github.com/mskcc/vcf2maf).

### TMB calculation

TMB is the total number of somatic mutations in exon regions, including point mutations and indels, usually measured per mega-base. We calculated the TMB for each sample using Maftools (2.12.05) (Mayakonda et al, 2018), with the capture length set to 50 Mbs by default. According to the cutoff defined in a previous study (Cancer Genome Atlas Research Network, 2014), samples with TMB values greater than 11.4 were labeled as high TMB (TMB-H), whereas others were labeled as low TMB (TMB-L). The TMB-H samples were hypermutated, and the TMB-L samples were regularly mutated.

### MSI detection

MSI, a condition in which the genome exhibits hypermutability because of DNA mismatch repair damage, is an important molecular phenotype in cancer. Information on microsatellite sequences was obtained from the reference genome using the scan module of MSIsensor-pro (v1.0.2) (Jia et al, 2020), and then, the calculation module was used to calculate the percentage of microsatellite sequences exhibiting MSI among all the microsatellite sequences. The percentage of the MSI sequences was defined as the MSI score. We calculated MSI scores for 185 patients. According to a systematic evaluation of multiple MSI calculation software (Bonneville et al, 2020), samples with MSI scores greater than 3.5% were considered to be MSI-H and others were MSI-L or MSS (microsatellite stable).

Because of the lack of gold-standard data for determining MSI from our NGS results, we also validated the MSI sites in the MSIsensor-pro results with the biomarkers used for MSI detection by PCR methods. The Bethesda panel (Boland et al, 1998) with two single-nucleotide repeat sites BAT-25 and BAT-26 and three dinucleotide repeat sites D2S123, D5S346, and D17S250 is commonly used to detect the MSI status. Samples that contain more than two biomarkers are labeled as MSI-H, and others are labeled as MSI-L/MSS. The kappa value was statistically used to determine the consistency of the two methods. The higher the kappa value, the higher the consistency. We calculated the kappa values of NGS results using three references and the Bethesda panel.

### Candidate driver gene detection

MutSig (v1.41) (Lawrence et al, 2013) was applied to evaluate whether genes are significantly mutated in a gastric tumor cohort using the algorithm MutSigCV. Compared with previous gene evaluation algorithms, MutSigCV adds covariates to the estimation of the background mutation rate for optimization. We took the somatic SNPs and indels detected in 166 TMB-L samples using the three reference genomes as input, calculated the significance of mutations in the cohort for the genes using MutSigCV (Lawrence et al, 2013) (the default parameter), and obtained the q-value for each gene after correction with FDR. Genes with q-value < 0.1 were candidate gastric cancer driver genes in the cohort. We counted the exon mutations in 185 patients of the candidate gastric cancer driver genes and concluded that genes with exon mutation rates greater than 5% in the population are most likely to be gastric cancer driver genes.

### Generation and analysis of simulation data

We got 39,605 structural variants in the construction of GGCPan. There are 3,632–4,682 structural variants (variant length more than 50 bps) in each sample. We randomly selected five samples from the 185 samples. There are 3,000–3,251 structural variants in the five samples. We simulated five whole-genome sequencing samples containing the structural variants of the five real samples using VarSim (Mu et al, 2015) (0.8.6-43-g74c4024). The simulated reads were paired-end with 30× depth and 150 bp long. We named this simulated data as SimuA. The rest of the parameters were default parameters. To evaluate the SV calling performance, the query SVs were compared with the ground truth set for each simulated sample using *truvari* (English et al, 2022) *bench* with options “*--multimatch -r 1000 -C 1000 -O 0.0 -p 0.0 -P 0.7 -s 50 -S 15 --sizemax 100000*.” In the calculation of precision, recall, and f1, the average of five samples per reference genome was taken.

### Generation of graph-modeled pangenomes with less samples

To evaluate the impact of the completeness of the graph-modeled pangenome on the detection of structural variants, we randomly selected 5, 10, 50, 110, and 150 samples from the 185 gastric cancer samples (excluding the five samples used for SimuA dataset) and constructed five graph pangenomes with these samples, respectively (see the Materials and Methods section for more details). The GGCPan was constructed with all the 185 gastric cancer samples. We got six different graph-modeled pangenomes. Then, we aligned SimuA reads to the six graph-modeled pangenomes and detected structural variants.

### Functional enrichment analysis

All the functional enrichment analysis was performed with Metascape (Zhou et al, 2019). The significance threshold was set to *P*-value < 0.05.

### Subtyping of 185 gastric cancers

Samples with the EBV-positive phenotype were firstly classified as EBV subtype. Then, the remaining samples that were labeled as MSI-H were classified as MSI subtype. The determination of GS and CIN was consistent with the determination method in TCGA article, which firstly used CNVkit (Talevich et al, 2016) and GISTIC2.0 (Mermel et al, 2011) to detect the somatic copy-number alteration (SCNA) of each sample, and then clustered the samples into two classes using hierarchical clustering methods (Euclidean distance, Ward’s method).

## Supplementary Material

Reviewer comments

## Data Availability

The sequencing data in this article have been deposited in the Genome Sequence Archive in National Genomics Data Center, China National Center for Bioinformation (GSA-Human), HRA002344 for normal gastric mucosa and HRA002333 for gastric cancer. The codes and somatic structural variants for this study are available at dudududu12138/GGCPan (github.com).
