# Supplementary material for: Gastric cancer genomics study using reference human pangenomes
Source: Life Sci Alliance. 2025 Jan 27;8(4):e202402977. doi: 10.26508/lsa.202402977 (PMC11772497; doi:10.26508/lsa.202402977)
Supplement: Supplementary file 1 [file LSA-2024-02977_Supplemental_Data_1.docx]

**Supplementary file for**

**Gastric cancer genomics study using reference human pangenomes**

Du Jiao, Xiaorui Dong, Shiyu Fan, Xinyi Liu, Yingyan Yu, Chaochun Wei

This file contains detailed information about evaluation of different tools to detect structural variants using three different references and manual check of 4 driver genes detected only using GGCPan(supplementary note 1 and 2), 7 supplementary figures and 6 supplementary tables (Supplementary Table 2-7). Supplementary Table 1 is in a separate excel file.

**Supplementary Notes**

**Supplementary Note 1** **Evaluation of the impact of different aspects on structural variant detection, including** **different reference genomes, structural variation identification methods, and the whole genome sequencing depths.**

There are three reference genomes used in this paper, two of which are linear reference genomes and one is a graph-modeled reference genome. Therefore, when evaluating the performance difference among them, two aspects are involved, one is the difference in the topology of the reference genomes (i.e., linear or graph), and the other is the difference caused by different SV identification tools. In addition, different sequencing depths may also have an impact on the results. We would like to assess how much impact the above 3 aspects have on the results.

We first evaluated the differences between different sequencing depths and variant detection tools. Kosugi et al systematically evaluated 69 tools for detecting structural variants based on whole-genome sequencing data(Kosugi et al. 2019), and we chose three tools that were well evaluated, efficient, and still available: SVaBa(Wala et al. 2018), Delly(Rausch et al. 2012), and Manta(Chen et al. 2016). We used these three tools to detect structural variants based on the GRCh38 (the linear reference genome) and used Survivor to merge the results of the three tools. The detection of structural variants in GGCPan is then done using vg. We extracted the autosomal SVs contained in GGCPan. There are 30,551 SVs. We simulated paired reads (150 bp) of 30×, 40×, and 50× sequencing depths containing these 30,551 SVs, with 3 samples per sequencing depth. Next, we aligned the reads from these 9 samples to GRCh38 and GGCPan and detected structural variants separately. We found that the precision and recall of GGCPan-based structural variant detection are both above 90%, far above the performance of the pipeline using GRCh38 as the reference (Supplementary Fig. 2a). As for the GRCh38-based structural variant detection, Manta and Delly still maintain a respectable precision, but the recall is much lower, even less than 50%, which means that matching short reads with linear genomes to detect structural variants inevitably misses a large proportion of true-positive results. The precision and recall of SvABA are both very low. The results of this study evaluating Manta, Delly, and SvABA are consistent with those derived in previous study(Kosugi et al. 2019). In addition, we used Survivor to merge the detection results of the 3 tools. We kept the variants detected in more than two tools and then evaluated the merged variants. Survivor's merged results only weakly improved the precision of the structural variants, but also discarded more true-positive SVs (Survivor's recall was lower than that of Manta and Delly, Supplementary Fig. 2a). Based on this evaluation, we finally used Manta to identify structural variants based on linear reference genomes. In addition, different sequencing depths had little effect on these tools.

In the simulation experiments described above, the structural variants used to construct the graph pan-genome and those used for evaluation are the same set of variants, thus it may be unfair to the linear model for performance comparison. The necessity to construct a population-specific reference genome is also unclear before this study. We therefore performed a new evaluation using real data. The GIAB project conducted a comprehensive study of the HG002 sample and released a reliable variant collection for researchers to use as a benchmark(Zook et al. 2020). The variant set includes 5156 deletions and 6850 insertions. We downloaded the sequencing data of HG002 sample (30×, Novaseq6000, 150bp, PCR free). We also downloaded the MinigraphCactus graph pangenome(Hicktey et al. 2024), which was constructed using 44 de novo assembled samples from 1,000 genomes project and GRCh38 and CHM13, excluding the HG002 sample. We aligned the sequencing data of HG002 sample to GRCh38 and MinigraphCactus graph pangenome, respectively. We used different pipelines to identify structural variants (Supplementary Figure 2c). We listed three pipelines for structural variation detection, which were named linear (directly align to GRCh38 and perform variant identification), graph2linear (remap the graph-based alignment back to GRCh38 and then perform variant identification), and graph (directly use the graph-based alignment for variant identification). Supplementary Figure 2d illustrate the evaluation results. As we can see, both the reference genome and the choice of tools have impact on the results. We can roughly assume that linear and graph2linear pipelines use the same tools, and we can see that both have similar precision. But the recall of graph2linear is lower than that of linear, which we attribute to the loss of reads during the“surject”process, and these reads mostly represent insertions. Graph and graph2linear pipelines use the same reference genome. Similarly, the recall of graph is overwhelming in recognizing insertions. Because using the graph process, the advantages of the graph model are preserved and reads are not discarded. However, we also see that the precision of the graph pipeline is greatly reduced because MinigraphCactus graph pangenome was constructed without including HG002, and the variants in the MC graph may not intersect as much with the true set of variants in the GIAB-identified samples, resulting in a particularly low precision. In the article published by the VG team(Hickey et al. 2020), the GIAB graph pangenome was constructed using the variants in HG002, and the performance of variant detection was evaluated using short reads from this sample (same as Supplementary Fig. 2c). The accuracy of is greatly improved when using the graph model constructed for this sample, where the insertion accuracy is improved to 0.649 (Supplementary Table. 7), reaching the same level as linear in the results of this experiment (Supplementary Fig. 2d). This suggests that both the choice of reference genome and the choice of tools have an impact and that it is necessary to construct population-specific pangenomes. This is particularly true in the field of disease, where disease samples will contain a number of specific pathogenic variants.We detected SVs using Manta with options:

*configManta.py --normalBam $normal_bam --tumorBam $tumor_bam --referenceFasta $reference --runDir $out/$sample/result*

*$out/$sample/result/runWorkflow.py -m local -j 16*

We detected SVs using Delly with options:

*delly call -x $excl -o $sample.bcf -g $reference $tumor-bam $normal-bam*

*delly filter -p -f somatic -o $sample-somatic.bcf -s sample.tsv $sample.bcf*

We detected SVs using SVaBa with options:

*$svaba run -t $bam -p 6 -D $known_indel -a $sample -G $reference*

We merged SVs from Manta, Delly and SVaBa using Survivor with options:

*$survivor merge vcf_files 1000 2 1 1 0 30 $sample.merged.vcf*

The link to download the structural variant benchmark for this sample provided by GIAB is <https://ftp.ncbi.nlm.nih.gov/pub/dbVar/data/Homo_sapiens/by_study/vcf/nstd175.GRCh38.variant_call.vcf> .

The link to download sequencing data of this sample is <https://ftp.1000genomes.ebi.ac.uk/vol1/ftp/data_collections/HGSVC2/working/20200805_NYGC_NA24385_Illumina_data/>.
