# Supplementary material for: Gastric cancer genomics study using reference human pangenomes
Source: Life Sci Alliance. 2025 Jan 27;8(4):e202402977. doi: 10.26508/lsa.202402977 (PMC11772497; doi:10.26508/lsa.202402977)
Supplement: Supplementary file 6 [file LSA-2024-02977_Supplemental_Data_2.docx]

**Supplementary file for**

**Gastric cancer genomics study using reference human pangenomes**

Du Jiao, Xiaorui Dong, Shiyu Fan, Xinyi Liu, Yingyan Yu, Chaochun Wei

This file contains detailed information about evaluation of different tools to detect structural variants using three different references and manual check of 4 driver genes detected only using GGCPan(supplementary note 1 and 2), 7 supplementary figures and 6 supplementary tables (Supplementary Table 2-7). Supplementary Table 1 is in a separate excel file.

**Supplementary Notes**

**Supplementary Note 2. Manual check of the 4 driver genes detected only by using GGCPan**

MutSigCV considers whether the target gene contains a silent mutation when determining whether a gene is a driver gene. If a gene has a higher percentage of non-silent mutations, it has a higher probability of being determined as a driver gene. Based on this, we checked the output reports of MutSigCV (Supplementary Table 6). It can be seen that the percentage of non-synonymous mutations identified using GGCPan is the highest (highlight in red). So, when using q<0.1 as a threshold, these 4 genes were identified.

The 5th gene, *MUC17*, was only identified in GCPan. We also checked the q values of this gene on the other two reference genomes and we found that the *MUC17* gene was at the edge of the threshold with q=0.26 and p=0 in GGCPan.
